# Cornea biomechanics and biometric evaluations in immunoglobulin G4-related ophthalmic disease

**DOI:** 10.1371/journal.pone.0347998

**Published:** 2026-05-20

**Authors:** Kenneth Ka Hei Lai, Fatema Mohamed Ali Abdulla Aljufairi, Jake Uy Sebastian, Xulin Liao, Wanxue Chen, Zhichao Hu, Yingying Wei, Ruofan Jia, Cheuk Wah, Andy Chi On Cheng, Joyce Kar Yee Chin, Andrew KT Kuk, Chi Ho Kwong, Nelson Kwok Foo Yip, Wendy Lam, Kenneth Kai Wai Li, Ngai Yang, Wilson Wai Kuen Yip, Alvin Lerrmann Young, Edwin Chan, Callie Ka Li Ko, Carmen Ka Man Chan, Hunter Kwok Lai Yuen, Li Jia Chen, Clement Chee Yung Tham, Chi Pui Pang, Kelvin Kam Lung Chong

**Affiliations:** 1 Department of Ophthalmology and Visual Sciences, Prince of Wales Hospital, Hong Kong Special Administrative Region, Hong Kong, China; 2 Department of Ophthalmology and Visual Sciences, The Chinese University of Hong Kong, Hong Kong Special Administrative Region, Hong Kong, China; 3 Department of Ophthalmology, Salmaniya Medical Complex, Government Hospitals, Manama, Bahsrain; 4 Department of Ophthalmology, Vicente Sotto Memorial Medical Center, Cebu City, Philippines; 5 Department of Statistics, The Chinese University of Hong Kong, Hong Kong Special Administrative Region, Hong Kong, China; 6 Department of Pathology, Queen Elizebeth Hospital, Hong Kong Special Administrative Region, Hong Kong, China; 7 Department of Ophthalmology, Hong Kong Sanatorium & Hospital, Hong Kong Special Administrative Region, Hong Kong, China; 8 Department of Ophthalmology, Tung Wah Eastern Hospital, Hong Kong Special Administrative Region, Hong Kong, China; 9 Department of Ophthalmology, Grantham Hospital, Hong Kong Special Administrative Region, Hong Kong, China; 10 Department of Ophthalmology, Caritas Medical Center, Hong Kong Special Administrative Region, Hong Kong, China; 11 Department of Ophthalmology, Tuen Mun Hospital, Hong Kong Special Administrative Region, Hong Kong, China; 12 Department of Ophthalmology, United Christian Hospital, Hong Kong Special Administrative Region, Hong Kong, China; 13 Hong Kong Eye Hospital, Hong Kong Special Administrative Region, Hong Kong, China; Public Library of Science, UNITED KINGDOM OF GREAT BRITAIN AND NORTHERN IRELAND

## Abstract

**Purpose:**

To evaluate the corneal biomechanical and biometric properties in immunoglobulin G4-related ophthalmic disease (IgG4-ROD) patients.

**Setting:**

Ethnic Han Chinese biopsy-proven IgG4-ROD patients from a territory wide cohort and sex-, age-, smoking- and lens status-matched healthy controls.

**Design:**

A case-controlled (1:1) study.

**Methods:**

Corvis ST analyzer and IOL Master 700 biometry assessment.

**Results:**

Sixty-seven eyes from 67 patients with IgG4-ROD (41 = males), aged 63.9 ± 13.1 years and 67 right eyes from 67 healthy ethnically Han Chinese controls were recruited. IgG4-ROD eyes were associated with a more negative applanation 2 velocity (−0.30 versus −0.27 m/s, *P* = 0.0143), and reduced radius of curvature (7.48 versus 7.98 mm, *P* = 0.0136). There was no difference in intraocular pressure, applanation 1 length, applanation 1 velocity, applanation 2 length, deformation amplitude and peak distance. Corneal astigmatism was greater in IgG4-ROD eyes (−1.2 versus −0.9 D*, P* = 0.0132), and the deep axis is along 90.2 degree. There is no difference in the steep and flat keratometry, deep axis, lens thickness, cornea thickness, and anterior chamber depth.

**Conclusions:**

We found multiple corneal changes in biomechanics and fine structures in a cohort of biopsy-proven IgG4-ROD with potential long-term implications in ocular surface functions. A longitudinal follow-up would be useful to identify the clinical implications and observe if these changes improve with treatments.

## Introduction

Immunoglobulin G4-related disease (IgG4-RD) is an immune-mediated systemic disease that was first recognized in a group of autoimmune pancreatitis (AIP) patients with elevated serum IgG4 levels in 2001 [1,2]. IgG4-RD is characterized by prominent IgG4+ plasma cells infiltration in multiple organs [3]. Immunoglobulin G4- related ophthalmic disease (IgG4-ROD) was first found in patients with sclerosing dacryoadenitis, and subsequent reports showed that it can also affect the extraocular muscles, cranial nerve branches, optic nerves, eyelid, periorbital and orbital soft tissue [4–6]. Organ-threatening complications included optic neuropathy and malignant changes were reported [7,8]. The pathogenic role of Immunoglobulin G4 antibodies is unclear, but the efficacy of B-cell depleting agents, e.g., rituximab, showed promising results that may signify the critical role of B cells in the IgG4-RD pathogenesis [9,10].

Each ocular adnexa has certain tension within the closed spherical organ, and the cornea is a transparent, dome-shaped structure that forms the front surface bordered by the eyelid and conjunctiva [11]. Biomechanics refers to the structural and mechanical properties of the cornea and is altered in different corneal or systemic disorders such as keratoconus, rheumatoid arthritis, and diabetes mellitus [11–14].

We notice that orbital involvement had been found in up to 95% of IgG4-ROD patients, and could corneal topographic changes were observed in other orbital disorders [5,15]. While structural changes of the cornea such as reduced nerve density and fiber length have been reported in IgG4-ROD [16], there has been no investigation of the biomechanical or biometric properties of the cornea in these patients. Previous studies in other orbital diseases have revealed characteristic corneal changes due to orbital inflammation and mechanical stress. However, whether IgG4-ROD affects the cornea in similar or distinct ways remains unknown. In this study, we hypothesize that there are corneal biomechanics, and biometric changes in IgG4-ROD patients.

## Methods

We conducted a case-control study including 67 most clinically manifested eyes of 67 biopsy-proven IgG4-ROD patients and 67 right eyes of 67 age-, sex-, smoking- and lens status-matched healthy subjects. IgG4-ROD patients with data collected for analysis in this study were recruited from a territory wide IgG4-ROD cohort between 3/7/2022 and 18/12/2022. Healthy subjects were recruited from a community eye screening program at the Chinese University of Hong Kong between 3/7/2022 and 18/12/2022. The IgG4-ROD territory wide cohort included patients, diagnosed with IgG4-ROD from 2005 to 2022, managed by hospitals of the Hospital Authority providing the publicly funded ophthalmology service in Hong Kong [5,17]. This study was approved by the Institutional Review Board as following: Joint Chinese University of Hong Kong- New Territories East Cluster Ethics Committee: 2020.478, New Territories West Cluster Ethics Committee: NTWC/CREC/17097, Kowloon Central Kowloon East Clusters Ethics Committee: KC/KE-17-164/ER-2, Kowloon West Cluster Ethics Committee: KW/EX-17-149(117-16), Hong Kong East Cluster Ethics Committee: HKECREC-2017-75, Hong Kong West Cluster Ethics Committee: UW18-120, and Hong Kong Sanatorium & hospital Ethics Committee: RC-2017-16. Written informed consent was obtained from every individual participant, and all study procedures conformed to the tenets of the Declaration of Helsinki.

Diagnosis of IgG4-ROD requires histological evidence of lymphoplasmacytic infiltrates of >50 IgG4+ cells per high power field (PHF) x400 and an IgG4+/ IgG+ ratio of >40% in the biopsied orbital tissue [18–20]. Orbital adnexal specimens from the 67 recruited IgG4-ROD patients including the lacrimal glands (n = 71), discrete masses (n = 34), infiltrated fat (n = 5), conjunctiva (n = 3), and extraocular muscles (n = 3) were obtained by open surgical biopsies. All the biopsies were conducted between January 2005 and December 2022 across participating study sites. In the present study, histopathological findings from these biopsies were used to confirm the diagnosis of IgG4-ROD. No further analysis of the biopsy specimens was performed. Patients with the following that may confound the result including a history of ocular inflammatory disease, neoplastic disease, glaucoma, or who had received ocular treatment including surgery, topical eyedrops, or contact lenses in the past 12 months were excluded from this study.

Corneal biomechanical parameters were measured using the Corvis ST analyzer (Corneal visualization scheimpflug Technology, Oculus; Wetzlar, Germany) by trained research assistants between 9 am and 4 pm. Patients were instructed to blink their eyes to achieve regular tear film before the examination. We included only measurements with quality scores of “OK”. The corneal biomechanical parameters were calculated and obtained using the built-in software. The corneal biometric parameters were measured by IOL Master 700 (Carl Zeiss Meditec AG, Jena, Germany). At least three measurements were obtained with axial length differences less than <0.05mm by the trained research assistants. All Corvis ST and IOLMaster 700 measurements were conducted between 9 am and 4 pm. The following biomechanical parameters were measured using the Corvis ST analyzer: intraocular pressure, applanation length 1 and 2, applanation velocity 1 and 2, maximum deformation amplitude, peak distance, and radius of curvature at the time of maximum concavity. Biometric parameters obtained from the IOLMaster 700 included lens thickness, steep and flat keratometry, corneal astigmatism, deep axis (corneal meridian), central corneal thickness, and anterior chamber depth.

Numerical results were presented as mean ± standard deviation (SD) and range unless otherwise stated. The comparison of corneal parameters between IgG4-ROD patients and healthy subjects was measured using the independent samples t-test or Mann-Whitney tests. We identified the clinically manifested eye in IgG4-ROD patients and the right eye of the healthy control subjects to avoid inter-eye correlation. The correlations between corneal and IgG4-ROD disease parameters were measured using Spearman correlation, taking *P* < 0.05 as the significant threshold. Receiver operating characteristics curve analysis was conducted to evaluate the accuracy of parameters for predicting IgG4-ROD patients from healthy subjects. All statistical analyses were performed using SPSS statistical software package (Windows version 24.0; IBM corporation., Armonk, NY).

## Results

Sixty-seven eyes from 67 ethnically Han Chinese patients with IgG4-ROD (41 = males), aged 63.8 ± 12.2 years, and 67 eyes from 67 age-, sex-, smoking- and lens status matched healthy ethnically Han Chinese controls were recruited. The smoking status, systemic comorbidities (e.g., hypertension, diabetes), ocular lens status, and axial length were comparable between the two groups (Table 1). Among the 67 patients with IgG4-ROD, 56 (84%) received systemic corticosteroids at some point.

**Table 1:** Demographic and clinical information of the IgG4-ROD patients and Healthy controls.

	IgG4-ROD	Healthy controls	P
No. patients	67	67	N/A
No. eyes	67	67	N/A
Age (years)	63.8 ± 12.2	63.3 ± 5.9	0.378
Male: Female	41:26	41:26	1.0
Ex/current smoker (N, %)	27%	21%	0.418
**Systemic disease**			
Hypertension (N, %)	22%	28%	0.799
Diabetes mellitus (N, %)	19%	13%	0.350
Dyslipidaemia (N, %)	20%	20%	1.0
**Ocular condition:**			
Pseudophakia(N, %)	12(18%)	12(18%)	1.0
Axial length (mm)	23.9	24.0	0.338

Abbreviations: IgG4-ROD = Immunoglobulin G4 related ophthalmic disease, mm = millimetre, No. = Number of, N/A Not applicable.

Regarding corneal biomechanics, IgG4-ROD eyes were associated with a more negative applanation 2 velocity (−0.30 versus −0.27 m/s, *P* = 0.0143), and reduced radius of curvature (7.48 versus 7.98 mm, *P* = 0.0136). There was no difference in intraocular pressure, applanation 1 length, applanation 1 velocity, applanation 2 length, deformation amplitude and peak distance (Table 2).

**Table 2:** The corneal biomechanical differences between IgG4-ROD patients and healthy control subjects.

	IgG4-ROD patients	healthy controls	*P* Value
Number of eyes	67	67	N/A
Intraocular pressure (mmHg)	15.3 ± 2.3	15.6 ± 2.5	0.290551
Applanation length 1 (mm)	2.11 ± 0.3	2.18 ± 0.3	0.134374
Applanation velocity 1 (m/s)	0.16 ± 0.02	0.17 ± 0.02	0.303392
Applanation length 2 (mm)	2.01 ± 0.3	2.05 ± 0.4	0.280556
Applanation velocity 2 (m/s)	−0.30 ± 0.04	−0.27 ± 0.06	0.014271*
Deformation amplitude (mm)	1.25 ± 0.6	1.15 ± 0.1	0.083253
Peak distance (mm)	5.01 ± 0.3	5.03 ± 0.3	0.138822
Curvature radius at the time of maximum concavity (mm)	7.48 ± 1.2	7.98 ± 0.9	0.01364*

Abbreviations: IgG4-ROD = Immunoglobulin G4 related ophthalmic disease, mm = millimetre, m/s = meter per second, mmHg = millimetres of mercury, N/A Not applicable.

*P<0.05

Corneal astigmatism was greater in IgG4-ROD eyes (−1.2 versus −0.9D, *P* = 0.0300). There is no difference in the steep and flat keratometry, deep axis, lens thickness, cornea thickness, and anterior chamber depth (Table 3).

**Table 3:** Differences in the IOL master parameters between IgG4-ROD patients and healthy control subjects.

	IgG4-ROD patients	healthy controls	*P* Value
Number of eyes	67	67	N/A
Astigmatism (D)	−1.2 ± 1.0	−0.9 ± 0.7	0.030*
Deep axis (degree)	90.2 ± 57.8	99 ± 46.5	0.150
Keratometry 2 (D)	44. 4 ± 1.5	44.3 ± 1.4	0.308
Keratometry 1 (D)	43.3 ± 1.4	43.4 ± 1.3	0.246
Central corneal thickness (um)	546.5 ± 30.3	550.2 ± 35.9	0.070
Anterior chamber Depth (mm)	3.2 ± 0.7	3.33 ± 0.7	0.224
Lens thickness (mm)	4.11 ± 1.4	3.86 ± 1.6	0.179

Abbreviations: D = Dioptres, IgG4-ROD = Immunoglobulin G4 related ophthalmic disease, mm = millimetre, N/A Not applicable. µm = micrometre,

*P<0.05

## Discussions

In this study, we identified multiple corneal changes in IgG4-ROD patients. The key biomechanical changes include a more negative applanation 2 velocity and a reduced radius of curvature at the highest concavity. Biometric analysis revealed an increase of with-the-rules corneal astigmatism in the cornea of IgG4-ROD. Structural changes of the cornea in IgG4-ROD were reported including both the lower nerve density and nerve fiber length [16], and the current study identifies further changes in the corneal biomechanics, and biometric properties.

Corneal biomechanical properties had been proposed as surrogate biomarkers for eye diseases. For example, a low corneal hysteresis was associated with an increased risk of progression in glaucoma patients [21–23]. The changes in the ability of the cornea to dissipate energy were observed following cataract surgery with cornea incisions or progressive corneal ectasia [24,25]. Applanation 2 velocity shows how fast the cornea return to the resting position following the second applanation. It has been suggested to be an indicator of cornea elasticity [26–28]. However, corneal elasticity cannot be measured with Corvis ST. Results of our study showed that IgG4-ROD corneas were also associated with a reduced area of concavity which implies a stiffer and less deformable cornea. Vellara et al reported that thyroid eye disease was associated with a more negative A2 velocity and a normal A1 velocity which is compatible with our data, and suggested an increased orbital content rigidity as the globe returns to the resting position at a faster rate [29]. The pathogenesis of the corneal biomechanical changes in IgG4-ROD patients is unclear, both mechanical and immunological factors should be investigated.

We would like to discuss the following interesting observation. Although IgG4-ROD eyes demonstrated a significantly reduced radius of curvature at the time of maximum concavity, there was no significant difference in keratometry values measured by the IOLMaster 700. The keratometry measurements reflect the static anterior corneal curvature under normal intraocular pressure. In contrast, the radius of curvature at the time of maximum concavity reflects the dynamic behavior of the cornea when subjected to an external air puff. A smaller radius of curvature at maximum concavity implies a stiffer, less deformable cornea, which may not necessarily manifest as a difference in the resting anterior curvature. Therefore, the dynamic parameters provided by Corvis ST may serve as more sensitive indicators of subtle corneal changes in IgG4-ROD. We noticed that the lens thickness was numerically greater in IgG4-ROD (4.11 mm versus 3.86 mm), despite statistically significant. One possible explanation is that prolonged corticosteroid use, which is common in IgG4-ROD, may accelerate age-related lens changes. We have ensured the lens status was matched for in both groups.

Thyroid eye disease is an autoimmune inflammatory orbital disease, and inflammatory cytokines were found in the tear sample that could lead to corneal stromal destruction [30,31]. The chemokine and cytokine profiles in the tear sample of IgG4-ROD were recently reported that were characterized by a distinct mixed immune response (Th1/ Th2/ Th17) [32]. Ocular surface and corneal endothelium change is reported in IgG4-ROD, future studies on the corneal damages from tear secretion of IgG4-ROD are warranted [33,34]. In addition, the cornea is bordered by the eyelid and sclera. Profound eyelid inflammation is commonly observed in IgG4-ROD patients. A longitudinal study to observe the corneal biomechanical changes following treatment is needed to understand the effects of mechanical stress on the ocular involvement in IgG4-ROD.

In the current study, IgG4-ROD patients were associated with higher with-the-rule astigmatism. Corneal biomechanical changes in keratoconus compromise the structural stability and weaken the mechanical strength which results in biometric changes [35]. However in healthy young subjects, the spherical aberration and corneal biomechanics are not related [36]. The pressure from the eyelid affects corneal astigmatism as demonstrated by the change of rules following a reduced pressure with age [37]. Thyroid eye disease was associated with greater with-the-rule-corneal astigmatism, and mechanical causes may be transmitted from sclera derived from extraocular muscle tension [38]. Flattening of the superior cornea and steepening of the inferior cornea had been reported in thyroid eye disease patients after inferior rectus muscle recession surgery [39]. In the current study, all 67 IgG4-ROD patients presented with upper eyelid inflammation, and 37% of patients in our territory-wide cohort had at least 1 extraocular muscle enlargement [5]. A longitudinal study to monitor the biometric changes following treatment is needed. To confirm the relationship between mechanical causes of IgG4-ROD and corneal astigmatism.

A major strength of our study is the inclusion of a relatively large, biopsy-proven cohort of IgG4-ROD patients, with strict matching to healthy controls for age, sex, smoking status, and lens status. To our knowledge, this is the first study to describe biomechanical and biometric changes in the cornea in IgG4-ROD, which could have diagnostic or prognostic implications. These findings provide a rationale for integrating corneal imaging into longitudinal studies, particularly to assess response to therapy. Despite its cross-sectional nature, the study lays important groundwork for future clinical trials and mechanistic investigations. The clinical implications of our findings lie in the potential use of corneal biomechanical properties as non-invasive biomarkers for subclinical orbital inflammation or fibrotic changes in IgG4-ROD. Future longitudinal studies incorporating treatment data and disease activity scores are warranted to validate the utility of these metrics in clinical practice.

There are several limitations in the current study. First, our patients were managed according to different management protocols at different collaborating centers. The accumulative steroid dose varied among them. Second, this is a cross-sectional study without follow-up data. Third, the structure and functional evaluation of the corneal such as confocal microscopy and corneal esthesiometer were not performed. Fourth, we acknowledge that disease activity and serum IgG4 levels were not assessed in this study, which limits our ability to correlate corneal biomechanical findings with disease severity or treatment response. This was due to the known inconsistencies in using serum IgG4 levels as a reliable marker of disease activity. Fifth, 95% of patients had lacrimal gland enlargement, making it the predominant site of ocular adnexal involvement. Although subgroup analysis by anatomical site could provide additional insights, the low frequency of isolated non-lacrimal gland involvement limited the feasibility of such an analysis. Sixth, associations that may affect the degree of corneal parameters such as lacrimal gland dimension, corneal sensation, and types and sites of ocular adnexal biopsies were not evaluated. Our data may serve as a baseline for future prospective cohorts or clinical trials to study the long-term characteristics of corneal changes in IgG4-ROD patients.

We report, for the first time, corneal changes including biomechanical and biometric properties in a cohort biopsy-proven IgG4-ROD. This may have long-term implications in ocular surface functions. A longitudinal follow-up study to show if these changes improve with treatments and/or over time and their visual implications is warranted.

### What was known:

- Structural changes of the cornea in IgG4-ROD included lower nerve density and nerve fiber length were reported.
- Both biomechanical and biometric changes were reported in other orbital diseases such as thyroid eye disease, yet, it was never studied IgG4-ROD patients.

### What this paper adds:

- IgG4-ROD eyes were associated with a more negative applanation 2 velocity and reduced radius of curvature when compared to healthy subjects.
- Corneal astigmatism was greater in IgG4-ROD eyes.
